# Simulations of radioiodine exposure and protective thyroid blocking in a new biokinetic model of the mother–fetus unit at different pregnancy ages

**DOI:** 10.1007/s00204-022-03331-0

**Published:** 2022-08-04

**Authors:** A. Rump, C. Hermann, A. Lamkowski, M. Abend, M. Port

**Affiliations:** Bundeswehr Institute of Radiobiology, Neuherberg Str. 11, 80937 Munich, Germany

**Keywords:** Medical NR protection, Nuclear and radiological emergency, Radioiodine, Thyroidal protection, Iodine blockade, Pregnancy, Mother–fetus unit

## Abstract

In the case of nuclear incidents, radioiodine may be released. After incorporation, it accumulates in the thyroid and enhances the risk of thyroidal dysfunctions and cancer occurrence by internal irradiation. Pregnant women and children are particularly vulnerable. Therefore, thyroidal protection by administering a large dose of stable (non-radioactive) iodine, blocking radioiodide uptake into the gland, is essential in these subpopulations. However, a quantitative estimation of the protection conferred to the maternal and fetal thyroids in the different stages of pregnancy is difficult. We departed from an established biokinetic model for radioiodine in pregnancy using first-order kinetics. As the uptake of iodide into the thyroid and several other tissues is mediated by a saturable active transport, we integrated an uptake mechanism described by a Michaelis–Menten kinetic. This permits simulating the competition between stable and radioactive iodide at the membrane carrier site, one of the protective mechanisms. The Wollf–Chaikoff effect, as the other protective mechanism, was simulated by adding a total net uptake block for iodide into the thyroid, becoming active when the gland is saturated with iodine. The model’s validity was confirmed by comparing predicted values with results from other models and sparse empirical data. According to our model, in the case of radioiodine exposure without thyroid blocking, the thyroid equivalent dose in the maternal gland increases about 45% within the first weeks of pregnancy to remain in the same range until term. Beginning in the 12th pregnancy week, the equivalent dose in the fetal thyroid disproportionately increases over time and amounts to three times the dose of the maternal gland at term. The maternal and fetal glands’ protection increases concomitantly with the amount of stable iodine administered to the mother simultaneously with acute radioiodine exposure. The dose–effect curves reflecting the combined thyroidal protection by the competition at the membrane carrier site and the Wolff–Chaikoff effect in the mother are characterized by a mean effective dose (ED_50_) of roughly 1.5 mg all over pregnancy. In the case of the fetal thyroid, the mean effective doses for thyroid blocking, taking into account only the competition at the carrier site are numerically lower than in the mother. Taking into account additionally the Wolff–Chaikoff effect, the dose–effect curves for thyroidal protection in the fetus show a shift to the left over time, with a mean effective dose of 12.9 mg in the 12th week of pregnancy decreasing to 0.5 mg at term. In any case, according to our model, the usually recommended dose of 100 mg stable iodine given at the time of acute radioiodine exposure confers a very high level of thyroidal protection to the maternal and fetal glands over pregnancy. For ethical reasons, the possibilities of experimental studies on thyroid blocking in pregnant women are extremely limited. Furthermore, results from animal studies are associated with the uncertainties related to the translation of the data to humans. Thus model-based simulations may be a valuable tool for better insight into the efficacy of thyroidal protection and improve preparedness planning for uncommon nuclear or radiological emergencies.

## Introduction

In nuclear fission reactions, uranium-235 usually splits asymmetrically and radioiodine (I-131) falls in one of the favored mass number regions of the fission products with a relatively high reaction yield. Moreover, iodine has a high volatility, so it is readily spread over larger distances (Chabot 2016). That is why it is a nuclide of particular concern in fallout resulting from a nuclear weapon detonation or a nuclear power plant accident. Iodine, and as it is chemically identical radioiodine, is rapidly absorbed into the blood after inhalation or ingestion. This applies to gaseous inorganic, gaseous organic or iodine adsorbed to particles (National Cancer Institute 2015). After absorption into the body, it is distributed in the extracellular space, eliminated through the kidneys and in parallel accumulates in the thyroid gland with an uptake mediated by an active carrier mechanism (iodide/sodium symporter, NI symporter in the basolateral membrane of the thyrocytes). The uptake fraction into the thyroid is usually in the range of 10–40% of the intake into the body, and 50% of this uptake occurs within 3–6.5 h (Kovary 1994; Geoffroy et al. 2000; Verger et al. 2001). However, the thyroidal uptake fraction shows a very high variability depending on nutritional iodine intake (Takamura et al. 2004; Reiners et al. 2013) as well as thyroid function (e.g., uptake fraction up to 80% in Grave’s hyperthyroidism) (Horn-Lodewyk et al. 2019). In the gland, iodide is oxidized and incorporated into tyrosyl residues of thyroglobulin, the precursor of the hormones triiodothyronine (T3) and thyroxine (T4), that is stored in the follicular lumen. The biological half-time of iodine in the thyroid gland, determined by the secretion of the hormones, is 90—100 days. In the case of (radio)iodine 131, there is an additional radioactive decay with a shorter half-life of 8.02 days leading to internal irradiation of the gland. This may lead to thyroidal dysfunctions (e.g., hypothyroidism by tissue destruction or induction of thyroiditis) and an enhanced occurrence of cancers (Reiners et al. 2020).

The thyroid can be protected against radioiodine by administering a large dose of stable (non-radioactive) iodine (100 mg) that competes with the radioactive entity at the NI symporter in the membrane and thus reduces radioactivity uptake into the gland (Reiners et al. 2013; Rump et al. 2016). Moreover, large amounts of iodine lead to a rapid inhibition of the inclusion of iodine into thyroglobulin, further reducing radioiodine accumulation in the thyroid (Wolff–Chaikoff effect) (Wolff et al. 1948; Geoffroy et al. 2000; Verger et al. 2001). This effect can best be imagined and described as a protective saturation mechanism. Although it is not yet fully understood, this effect could at least in part be mediated by the inhibition of peroxidase activity and iodide oxidation as the step preceding organification in tyrosyl residues (Bürgi 2010; Leung et al. 2014). The Wolff–Chaikoff effect is only transient and fades after 24–48 h (Geoffroy et al. 2000; Verger et al. 2001; Leung et al. 2014), and this must be considered in the case of prolonged accidental radioiodine exposure. During the period, the radioiodine uptake into the gland is inhibited, it is further eliminated through the kidney with a half-time of roughly 8 h (clearance between 30 and 50 ml/min) (Geoffroy et al. 2000). Renal elimination is not a saturable process and thus not inhibited by large stable iodine doses. When the Wolff–Chaikoff effect has faded and thyroidal iodide uptake is restored, most of the radioiodide in the body can be expected to have already been excreted. As the uptake of iodide from the blood into the gland is a rapid process, thyroid blocking must be achieved shortly before or within a few hours after radioiodine exposure (Geoffroy et al. 2000; Verger et al. 2001; ASN 2008; Rump et al. 2016; WHO 2017; SSK 2018). Administering stable iodine after more than 24 h after acute radioiodine exposure does not confer a relevant protective effect. According to the World Health Organization (WHO 2017), “provision of iodine thyroid blocking (ITB) to people who are at risk of being exposed to radioiodine should be implemented as an urgent protective action, within the frame of a justified and optimized protection strategy”. This recommendation is also part of many national guidelines for nuclear or radiological emergencies (e.g., France, Germany) (ASN 2008; SSK 2018). The recommended stable iodine dosages in the literature and guidelines amount to 100 mg iodine (corresponding to 130 mg potassium iodide) (e.g., in Germany) (SSK 2018) or in some countries 76 mg iodine (100 mg potassium iodide) (e.g., in Japan) (Yoshida et al., 2014).

A particular issue relates to thyroid blocking in vulnerable subpopulations like children or pregnant women. Children have higher risks to develop thyroid diseases and cancer after radioiodine exposure as the gland is smaller, the radioiodine uptake and absorbed radiological dose are higher and the tissues are more sensitive to radiation (Iglesias et al. 2017). The situation is particularly complex for pregnant women as iodine metabolism is physiologically altered (Aboul-Khair et al. 1964; Feely 1979): the renal clearance of iodide is increased to an extent not fully explained by the enhanced glomerular filtration; the extracellular space is expanded and thus the volume of distribution of iodide increased. There is, in addition, a loss of iodide from the mother to the fetus, and all these factors contribute to lower serum inorganic iodide concentrations in pregnancy, associated with an increase in the maternal thyroidal clearance of iodide. Iodide readily crosses the placenta (Verger et al. 2001; WHO 2017) and the fetal thyroid gland begins to concentrate it from the 11th to 12th week of pregnancy (post conception). At term, iodine concentration in the fetal thyroid is known to exceed several times the values in the maternal gland (Aboul-Khair et al. 1966; Evans et al. 1967; Stieve et al. 1985; Berkovski 2002). In the case of radioiodine exposure, the fetal thyroid is expected to absorb higher radiological doses than the maternal gland. Besides stochastic radiation damage, this may lead to fetal hypothyroidism. However, a sufficient level of thyroid hormones is of major importance for neurocognitive development. The transplacental transfer of thyroxine from the mother to the fetus beyond the first trimester is limited by placental deiodinases, whose activities increase with gestation (Girling 2003). Therefore, in the case of radioiodine exposure, thyroid blockade is very important not only for the mother but also for the fetus, which is acknowledged in the literature and in official guidelines (WHO 2017).

**Fig. 1:**
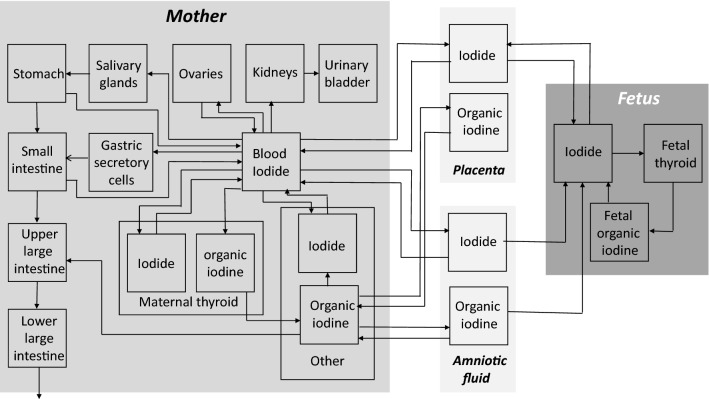
Compartment model for pregnant women with embryo/fetus (Berkovski 2002). All transport processes between compartments are described by first-order kinetics. Several rate constants of the exchange processes vary in the course of pregnancy (see Tables 2 and 3)

Assessing the risks from radioiodine exposure and the efficacy of thyroid blocking for the mother and fetus at different stages of pregnancy is a challenge. Equivalent doses absorbed by the tissues after incorporating a radionuclide cannot be measured like in the case of external irradiation. Still, they must be calculated based on biokinetic–dosimetric models (internal dosimetry). Specific models have been developed to describe radioiodine kinetics in pregnant women, including the embryo/fetus. The International Commission of Radioprotection has adopted the model of Berkovski (1999a, 1999b and 2002) (ICRP) (ICRP 2001). Although the model is much differentiated in its structure (Fig. 1), exchanges between compartments are described by first-order kinetics with fixed rate constants determined for various pregnancy ages. Therefore, it does not permit to simulate thyroid blocking effects. In the past, thyroid blocking in adults has been modeled by expressing the rate constant of iodide transport into the gland as a non-linear function of serum iodide concentration (Blum et al. 1967) or as a linear function of the iodine content of the gland relative to a saturation amount at which uptake stops (Adams et al. 1962; Ramsden et al. 1967). However, these models do not permit to differentiate between the relative contributions of competition at the NI symporter and the Wolff–Chaikoff effect. In newer models, thyroidal iodide uptake is described by saturable Michaelis–Menten kinetics and the Wolff–Chaikoff effect by an additional temporary uptake block once a saturation level has been reached, permitting to quantitatively differentiate both mechanisms (Rump et al. 2019 and 2021a; Eder et al. 2020). Similarly to this latter approach, we modified the pregnancy model of Berkovski by integrating saturable kinetics at the sites the NI symporter is known to mediate iodide transport and in addition, modeled the Wolff–Chaikoff effect using gland saturation levels. The new model was used to simulate the protective efficacy achieved by stable iodine against radioiodine in the mother and fetus at different pregnancy ages.

## Methods

### Development of a new biokinetic iodine model of the mother–fetus unit

#### Integration of an active carrier uptake mechanism into an established model

We departed from the mother–fetus biokinetic model for radioiodine established by Berkovski (2002) and adopted by the ICRP (2001, corrected version in 2002) (Fig. 1). Iodide transport into the thyroid, the salivary glands, the gastric mucosa (Nicola et al., 2009) and the placenta (Richard et al., 2012) are known to be mediated by the NI symporter. Therefore, as previously described (Rump et al. 2019 and 2021a; Eder et al. 2020), these transfer processes were modeled in analogy to an enzyme reaction by Michaelis–Menten kinetics:

$$T= \frac{Tmax *C}{Km + C},$$

T is the transport capacity through the membrane at the time when C is the concentration of iodide in the source compartment. The Michaelis–Menten constant (*K*_m_) and the maximum transport capacity (*T*_max_) are the parameters of the equation. For the human NI symporter, a *K*_m_ of 9 µmol.l^−1^ has been reported (Darouzet et al. 2014) and this value was used in our model for the mother and the fetus. *T*_max_ values for the different processes were derived from the *K*_m_ and the respective first-order rate constants *k*_i_ described in the model of Berkovski (2002). To express this parameter as an absolute amount per time unit (µmol d^−1^ and not µmol l^−1^ d^−1^), we entered the volume of distribution *V*_d_ of the source compartment into the equation: *T*_max_ = *K*_m_ * *k*_i_ * *V*_d_.

The intestinal uptake of iodide from the intestine is also mediated by the NI–symporter (Nicola et al., 2009). However, in our adapted model, these processes were not modeled by Michaelis–Menten kinetics, as it is impossible to realistically estimate the volumes of iodine distribution in the highly variable gastrointestinal content. The first-order kinetics with the rate constants given by Berkovski (2002) were kept for our computations.

For the volume of distribution of the blood compartment of the mother, we used the value of 7 l as given by Berkovski (2002) in his original model. We determined the total weight of the fetus from the literature (Kiserud et al. 2017), and taking into account that the extracellular space is relatively larger than in adults (Hall 2016), we assumed, like Berkovski (2002), that the volume of distribution for iodide in the fetus corresponds to 50% of its weight. The physiological values used in our model at different pregnancy ages are shown in Table 1. All the kinetic parameters used in our model (first-order rate constants adopted from Berkovski and the derived parameters for the Michaelis–Menten kinetics) are displayed in Tables 2, 3 and 4. This paper gives pregnancy age as post-conceptional age (i.e., gestational age minus 2 weeks).

**Table 1:** Physiological parameters in pregnancy as used in our model to derive the parameters of the Michaelis–Menten equations (*V*_d_) or applied for dosimetric calculations (weight of the thyroid)

	Pregnancy week								
	0	3	8	12	16	24	32	36	38
	Mother								
*V*_d_ blood (l)	7	7	7	7	7	7	7	7	7
Thyroid weight (g)	16.0	16.0	16.0	16.93	16.93	16.93	18.38	18.38	19.83
Lobe radius (cm)	1.270	1.270	1.270	1.294	1.294	1.294	1.330	1.330	1.364
	Fetus								
Fetal weight (g)	–	–	–	90	222	902	2,312	3,186	3,617
*V*_d_ fetus (l)	–	–	–	0.045	0.111	0.451	1,156	1,593	1,808.5
Thyroid weight (g)	–	–	–	0.101	0.166	0.475	1.05	1.25	1.30
Lobe radius (cm)	–	–	–	0.197	0.252	0.392	0.471	0.502	0.516

*V*_d_: iodide volume of distribution (for the mother blood compartment; for the fetus 50% of fetal weight). Source of the data: maternal thyroid weight and lobe radius derived from volume changes over pregnancy (Glinoer et al. 1990) with initial radius before pregnancy 1.27 cm (National Cancer Institute 2015); fetal body weight (Kiserud et al. 2017); fetal thyroid weight (Ratnakar Rao et al. 2015; Berkovski 2002); fetal lobe radius derived from thyroid volume (Barbosa et al. 2019)

**Table 2:** Parameters of the biokinetic model for iodine in pregnancy for the carrier-mediated processes (for the structure, see Figs. 1 and 2)

		Pregnancy week								
		0	3	8	12	16	24	32	36	38
		Mother								
Blood to thyroid iodide	*k*	2.3	2.3	2.3	2.3	2.3	2.3	2.3	2.3	2.3
	*K*_m_	63	63	63	63	63	63	63	63	63
	*T*_max_	144.9	144.9	144.9	144.9	144.9	144.9	144.9	144.9	144.9
Blood to thyroid organic iodine	*k*	2.3	5.1	6.5	7.5	8.4	9.3	9.1	8.8	8.7
	*K*_m_	63	63	63	63	63	63	63	63	63
	*T*_max_	144.9	321.3	409.5	472.5	529.2	585.9	573.3	554.4	548.1
Blood to salivary gland	*k*	3.0	3.0	3.0	3.0	3.0	3.0	3.0	3.0	3.0
	*K*_m_	63	63	63	63	63	63	63	63	63
	*T*_max_	189	189	189	189	189	189	189	189	189
Blood to gastric secretory cells	*k*	8.6	8.6	8.6	8.6	8.6	8.6	8.6	8.6	8.6
	*K*_m_	63	63	63	63	63	63	63	63	63
	*T*_max_	541.8	541.8	541.8	541.8	541.8	541.8	541.8	541.8	541.8
Blood to placenta iodide	*k*	0	0	33	33	33	33	33	33	33
	*K*_m_	–	–	63	63	63	63	63	63	63
	*T*_max_	–	–	2079	2079	2079	2079	2079	2079	2079
		Fetus								
Fetal iodide to fetal thyroid	*k*	0	0	0	1.8	3.5	5.0	6.0	6.0	6.0
	*K*_m_	–	–	–	0.405	0.999	4.059	10.404	14.337	16.2765
	*T*_max_	–	–	–	0.729	3.4965	20.295	62.424	86.022	97.659

The first-order kinetic rate constants (k in d^−1^) are from the Berkovski (2002) model. In our new model, the carrier-mediated transport processes are described by Michaelis–Menten kinetics with two parameters: the Michaelis–Menten constant (*K*_m_) expressed in µmol d^−1^ instead of concentration (µmol l^−1^) as we use iodine amounts (µmol) in our computations; *T*_max_ (µmol d^−1^): the maximum transport capacity of the process. For the derivation of *K*_m_ et *T*_max_ from the rate constants *k* and the volumes of distribution (Table 1), see the Method section

**Table 3:** Rate constants (d^−1^) of transport processes following first-order kinetics and dependent on pregnancy age as used in our new model

	Pregnancy week								
	0	3	8	12	16	24	32	36	38
Blood to kidneys	6.4	7.0	8.9	10.0	12.0	13.0	12.0	12.0	12.0
Blood to amniotic fluid iodide	0	0.75	0.75	0.75	0.75	0.75	0.75	0.75	0.75
Organic iodine in other to iodide in other	0.03	0.038	0.038	0.038	0.038	0.038	0.038	0.038	0.038
Organic iodine in other to upper large intestine	0.0074	0.0094	0.0094	0.0094	0.0094	0.0094	0.0094	0.0094	0.0094
Organic iodine in other to uteroplacental unit organic iodine	0	0	18	20	24	31	40	46	51
Organic iodine in other to amniotic fluid organic iodine	0	5.5	5.5	5.5	5.5	5.5	5.5	5.5	5.5
Uteroplacental unit iodide to blood	11,000	11,000	11,000	3,600	1,400	510	270	190	160
Uteroplacental unit iodide to fetal iodide	0	0	0	33	33	33	33	33	33
Uteroplacental unit organic iodine to organic iodine in other	18,000	18,000	18,000	7,600	3,900	1,900	1,500	1,500	1,600
Fetal iodide to uteroplacental unit iodide	0	0	0	100	60	35	20	15	12
Thyroid organic iodine to organic iodine in other	0.0063	0.013	0.013	0.013	0.013	0.013	0.013	0.013	0.013
Amniotic fluid iodide to blood	130	130	130	43	17	6	3.2	2.2	1.9
Amniotic fluid iodide to fetal iodide	0	0	0	0.25	0.25	0.25	0.25	0.25	0.25
Amniotic fluid organic iodine to organic iodine in other	6,400	6,400	6,400	2,300	990	380	230	200	190
Amniotic fluid organic iodine to fetal iodide	0	0	0	0.25	0.25	0.25	0.25	0.25	0.25

**Fig. 2:**
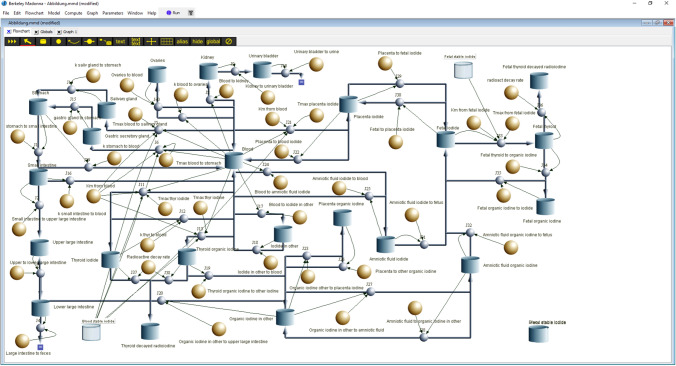
Flow chart of the new pregnancy model derived from the original model of Berkovski (2002) by integrating saturable carrier-mediated processes described by Michaelis–Menten kinetics with the parameters K_m_ and T_max_ at sites where the iodide transport is mediated by the NI symporter. The figure shows the compartment model for radioiodide. The compartment model must be doubled to simulate thyroid blocking by stable iodide. In the displayed flow chart, the compartments labeled "blood stable iodide" and "fetal stable iodide" are "alias compartments" representing the blood compartments for stable iodide of the mother and child, respectively. Their iodide contents inhibit the NI symporter-mediated transport processes

For the model structure, see Figs. 1 and 2. Source of the data: Berkovski (2002)

**Table 4:** Rate constants (d^−1^) of transport processes following first-order kinetics and remaining unchanged over pregnancy

Stomach to blood	40
Stomach to small intestine	24
Small intestine to blood	300
Small intestine to upper low intestine	6
Blood to iodide in other	48
Blood to thyroid iodide	2.3
Blood to ovaries	17
Iodide in other to blood	19
Thyroid iodide to blood	1.6
Ovaries to blood	590
Salivary glands to stomach	10
Gastric secretory cells to stomach	20
Kidneys to urinary bladder	10
Fetal thyroid to fetal organic iodine	0.035
Fetal organic iodine to fetal iodide	0.13
Upper large intestine to lower large intestine	1.8
Lower large intestine to feces	1
Urinary bladder to urine	12

For the model structure, see Figs. 1 and 2. Source of the data: Berkovski (2002)

#### Modeling thyroid blocking: competition at the carrier site

As previously described (Rump et al. 2019 and 2021a; Eder et al. 2020), to model the competition of stable iodide and radioiodide at the NI symporter, we applied the rate law for monomolecular irreversible enzyme reactions with two competing substrates (Chou et al. 1977; Schäuble et al. 2013):

$$T1= \frac{Tmax*C1}{Km1*\left(1 + \frac{C2}{Km2}\right)+C1},$$

with *T*_1_ the transport rate and *T*_max_ the maximum transport rate for substrate 1, *K*_m1_ and *K*_m2,_ the Michaelis–Menten constants for substrate 1 and 2, respectively (in our case *K*_m1_ = *K*_m2_ = 9 µmol.l^−1^, as the 2 competing entities radioiodide and stable iodide are chemically identical) and *C*_1_ and *C*_2_ the concentrations of substrate 1 and 2, respectively.

As in our computations, we used iodine amounts m (in µmol) and not concentrations in the compartments of our model. We multiplied the numerator and denominator of the equation with the distribution volume *V*_d_ of the source compartment. Thus, the equation finally used to describe the uptake of radioiodide into the thyroid in the presence of stable iodide using molar units was as follows:

$$ {\text{T}}_{{{\text{I}} - {131}}} = \left( {{\text{T}}_{{{\text{max}}}} *{\text{ m}}_{{{\text{I}} - {131}}} } \right) \, / \, \left( {{\text{K}}_{{\text{m}}}^{\# } + {\text{ m}}_{{{\text{I}} - {131}}} + {\text{ m}}_{{\text{I}}} } \right), $$

with *T*_I-131_ the transport rate for radioiodide (µmol d^−1^), *T*_max_ the maximum transport rate (µmol d^−1^), *K*_m_^#^ the Michaelis–Menten constant expressed as absolute amount (= *K*_m_ * *V*_d_) (µmol) and m_I-131_ and m_I_ the respective amounts of radioiodide and stable iodide (µmol) in the source compartment.

For transport processes following Michaelis–Menten kinetics, the source compartments, according to the structure of our model, are the blood compartment in the mother (the interstitial compartment is separate) and the total extracellular compartment in the fetus.

#### Modeling thyroid blocking: the Wolff–Chaikoff effect

To model the Wolff–Chaikoff effect, we added a saturation mechanism leading to a total net block of iodide uptake once the gland is saturated. It was reported that this total uptake block becomes effective when the iodine content of the adult gland increases by 350 µg (2.7581 µmol) (from 8,000 µg to 8,350 µg) (Riggs 1952; Ramsden et al. 1967), and that is the value we used for the maternal thyroid. The Wolff–Chaikoff effect is a temporary phenomenon lasting about 24–48 h and we assumed an escape 36 h after the start of the uptake block. Thus, we modeled the Wolff–Chaikoff effect, as previously, as a “switch on” and “switch off” mechanism (Rump et al. 2019 and 2021a; Eder et al. 2020).

As far as we know, iodine saturation amounts for the fetal thyroid have not been reported yet. We assumed that the Wolff–Chaikoff effect is switched on for the fetal thyroid when the additional iodine uptake corresponds to the same fraction of the iodine content as in the adult gland (+ (350 µg/8000 µg)*100% =  + 4.375%). Furthermore, as there are no quantitative values available, we also assumed that the fetal thyroid escapes the Wolff–Chaikoff effect (“switch off”) after 36 h, similarly to an adult thyroid.

For computations, the (radio)activities (Bq) were at first transformed in µmol using the known specific activity of iodine 131 (4.6*10^15^ Bq/g). All biokinetic computations were done with Berkeley Madonna software and the flow chart editor (Macey et al. 2009) (Fig. 2). As an integration method to determine the (radio)iodide amounts in the individual compartments, we selected the Rosenbrock integration method.

#### Calculation of the thyroid equivalent dose

Besides eliminating radioiodine from the thyroid compartment as iodide or organically bound iodine as biokinetic processes, we modeled the radioactive decay (physical *T*_1/2_ = 8.02 d) and quantified the decayed amount. In practice, this was done by adding in our model a sink where the decayed radioiodine in the maternal or fetal thyroid was cumulatively collected. From the collected amounts expressed in mol, we derived the number of entities that were decayed using the Avogadro constant (6.022*10^23^ entities/mol). Applying the mean energy of the emitted ß-radiation (0.18 MeV/decay), we computed the total energy due to ß radiation absorbed by the gland. Through division by the weight of the thyroid, we got the energy dose (mGy) that is numerically identical to the equivalent dose (mSv), as the quality factor for ß-radiation is unity.

Although in the case of iodine 131, most of the dose is caused by ß-radiation, there is a small additional amount by the emission of ɣ-radiation. To take into account this fraction, we used an equation based on the method of Marinelli/Quimby (Marinelli et al. 1948) that combines the contribution of the ß-radiation with the geometrical factor method of Hine et al. (1956) for the (low) contribution of the ɣ-radiation:

$$ D{\mkern 1mu} = {\mkern 1mu} C_{\max } *{\mkern 1mu} T_{eff} *{\mkern 1mu} \left( {73.8{\mkern 1mu} *{\mkern 1mu} {\overline{\text{E}}}{}_{{\upbeta }} + {\mkern 1mu} 0.0346{\mkern 1mu} *{\mkern 1mu} {\mathcal{T}}*{\mkern 1mu} \overline{g}} \right) $$

With D the total dose from ß- and ɣ-radiation (rad), *C*_max_ the maximum concentration of the radionuclide in tissue (µCi g^−1^), *T*_eff_ the effective half-life in the tissue (days) (7.3 days for I-131 in adults), *Ē*_ß_ the average beta energy (MeV per disintegration) (0.18 MeV for I-131), *Ƭ* the specific ɣ ray constant (R per mCi h^−1^ at 1 cm) (2.2 R mCi^−1^ h^−1^) and ḡ the average geometrical factor for the tissue or organ, equal to 3 π r for spheres with radii < 10 cm. For our purpose, only the sum in brackets is of interest: The quotient of the second term relative to the sum ((0.0346 * Ƭ * ḡ)/(73.8 * Ē_ß_ + 0.0346 * Ƭ * ḡ)) is the fraction of the total absorbed dose due to ɣ- radiation.

To calculate the equivalent dose absorbed, the weight of the gland and radius of a thyroidal lobe have to be known. The best estimate for the thyroid weight for a non-pregnant woman (i.e., pregnancy week 0) was assumed to be 16 g (National Cancer Institute 2015). It is known that the thyroid volume may increase over pregnancy, and the gland’s weight was corrected accordingly (Glinoer et al. 1990). For the fetal thyroid, we used the values given in the literature for the different ages of pregnancy (Ratnaka Rao et al. 2015). The physiological values used for dosimetric calculations at the different pregnancy ages are displayed in Table 1.

The average geometrical factor depends on the radius of a thyroidal lobe if approximated to a sphere. For the adult thyroid of a non-pregnant woman, we used *r* = 1.27 cm (National Cancer Institute 2015). The radius increase over pregnancy was derived from the volumes (Glinoer et al. 1990). For the fetal thyroid, the volumes of the gland over pregnancy given in the literature were used (Barbosa et al. 2019). Based on these values, we calculated the corresponding radii and the resulting geometrical factors (Table 1). For the maternal thyroid, ɣ-radiation contributes to the total absorbed dose by 6–7%. In the fetal thyroid with a smaller lobe radius, the contribution of gamma radiation is even less.

It should be emphasized that the radiological doses we calculated are equivalent doses (energy dose * quality factor of the radiation, for ß- and ɣ-radiation the quality factor = 1), and not effective doses additionally taking into account the radiation sensitivity of a tissue regarding stochastic health effects (effective dose = equivalent dose * tissue weighting factor, for the thyroid the sensitivity factor = 0.05, but equivalent and effective doses are both expressed with the same unit mSv).

## Application of the new model

### Validation of the new model

The equivalent thyroid doses for radioiodine intakes ranging from 100 to 10^15^ Bq were calculated using the original model of Berkovski and the modified new model. Although refined models have been proposed for ingestion or the inhalational pathways of iodine (Johansson et al. 2003; Harvey et al. 2004), to exclude effects related to the structure and parameters of the inhalational or gastrointestinal model, in our simulations, (radio)iodine was administered by injection directly into the blood compartment of the mother. We consider that this is legitimate as the absorption rate for iodine is rapid and the absorption extent almost complete (Geoffroy et al. 2000; Verger et al. 2001; ATDSR 2004; Rump et al. 2019). Similarly, in all further simulations of this analysis, radioiodine or stable iodine directly entered into the maternal blood compartment.

Furthermore, the equivalent doses absorbed by the maternal thyroid at week 0 of pregnancy (i.e., non-pregnant woman) were compared with the values obtained with the Integrated Modules for Bioassay Analysis (IMBA) software (Birchal et al. 2007). IMBA is a commercial software based on the biokinetic model of the ICRP for radioiodine derived from the model of Riggs (1952). Moreover, thyroid equivalent doses were compared to the values calculated with a simple 2 compartment model previously developed. A carrier mechanism was integrated with Michaelis–Menten kinetics for iodide uptake into the gland (Rump et al. 2019).

Iodine uptake fractions by human maternal and fetal thyroids have been reported in a few studies (Chapman et al. 1948; Hodges et al. 1955; Evans et al. 1967). Prior to abortions and in 3 cases of anencephalic fetuses at term, the mothers had been given iodine 131 and the uptake fraction was measured in the maternal and fetal glands. We compared the measured values with the values predicted by our model for the respective pregnancy week.

#### Maternal and fetal thyroid equivalent doses after acute radioiodine exposure without thyroid blocking

For our simulations, we used a radioiodine intake of 700,000 Bq that, based on IMBA calculations, led to an equivalent thyroid dose of roughly 300 mSv that has been set up as the maximum permissible level by German occupational regulations for non-pregnant adults. Using our new model, we computed the equivalent doses that the maternal and fetal thyroid would absorb at different weeks of pregnancy.

#### Estimation of the protective efficacy of different single doses of stable iodine

Different doses of stable iodine in a range of 0.1 mg to 1,000 mg were administered to the mother at the same time as radioiodine (700,000 Bq) and the protective efficacy was determined for the mother and fetus. The protective efficacy of the iodine blockade was determined as the complementary value of the dose reduction factor:

$$ {\text{Efficacy }} = { 1 }{-} \, \left( {{\text{thyroid dose with iodine blockade }}/{\text{ thyroid dose without iodine blockade}}} \right) $$

The dose–effect-relations were examined by fitting the data to a sigmoidal Hill equation $(\mathrm{E}=\frac{a*{D}^{b}}{{D50}^{b}+ {D}^{b}})$ and the median effective doses (ED_50_) were determined for the mother and fetus. Calculations were done considering only the competition at the carrier site or including the Wolff–Chaikoff effect. In the latter case, the time points at which saturation was achieved and the total net iodide uptake block initiated (Wolff–Chaikoff "switch on") were registered.

#### Estimation of thyroidal protection by the recommended stable iodine dose

The efficacy of the usually recommended dose of 100 mg stable iodine for protecting the maternal and fetal thyroid in the different stages of pregnancy was derived from the dose–effect curves. Besides total protective efficacy, the relative contributions of the competition at the carrier site in the membrane and the Wolff–Chaikoff effect were quantitated to be compared to previous simulation findings.

## Results

### Validation of the new model

**Fig. 3:**
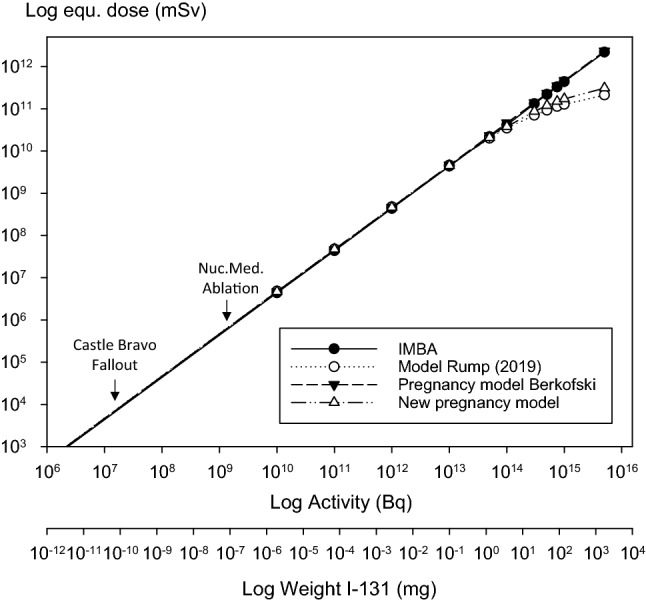
Thyroid equivalent doses (mSv) in non-pregnant women (= pregnancy week 0) resulting from different radioiodine intakes (Bq) as predicted by various models. Computations by IMBA are based on the ICRP model for radioiodine derived from Riggs (1952) model. The model of Rump et al. (2019) is derived from the ICRP model with the integration of a carrier-mediated uptake of iodide into the thyroid similar to the new pregnancy model introduced in the present study. Nuc.Med. Ablation: Range of activities used for post-surgical radioiodine ablation of residues in patients with thyroid cancer. Castle Bravo fallout: Activity incorporated on average by the victims of a nuclear weapon test accident with early fallout exposure on the Marshall Islands (code name “Castle Bravo”, March 1954)

The equivalent thyroid doses calculated using IMBA, the model of Berkovski for the pregnancy week 0, the simple model with integrated NI symporter to study thyroid blocking (Rump et al. 2019) or the adapted pregnancy model developed in this study are quite comparable up to radioiodine activities in the range of 10^14^ Bq (Fig. 3, Table 5). The equivalent dose corresponding to 10^14^ Bq amounts to roughly 4*10^10^ mSv. Results given by the original model of Berkovski for pregnancy week 0 (i.e., non-pregnant state) using first-order kinetics are numerically identical to the values calculated with our adapted model using Michaelis–Menten kinetics up to activities about 10^11^ Bq, as both models are equivalent for very low iodide concentrations.

**Table 5:** Comparison of thyroid equivalent doses (mSv) after the acute intake of different activities of iodine 131 calculated using different models

Activity (Bq)	IMBA	Model (Rump et al. 2019)	Pregnancy model (Berkovski 2002)	New pregnancy model
100	4.37 * 10^–2^	4.79 * 10^–2^	4.637 * 10^–2^	4.637 * 10^–2^
10,000	4.37	4.792	4.637	4.637
100,000	4.37 * 10^1^	4.7 * 10^1^	4.637 * 10^1^	4.637 * 10^1^
10^10^	4.37 * 10^6^	4.792 * 10^6^	4.637 * 10^6^	4.637 * 10^6^
10^11^	4.37 * 10^7^	4.790 * 10^7^	4.637 * 10^7^	4.637 * 10^7^
10^12^	4.37 * 10^8^	4.772 * 10^8^	4.637 * 10^8^	4.624 * 10^8^
10^13^	4.37 * 10^9^	4.601 * 10^9^	4.637 * 10^9^	4.520 * 10^9^
5 * 10^13^	2.19 * 10^10^	1.999 *10^10^	2.318 * 10^10^	2.072 * 10^10^
10^14^	4.13 * 10^10^	3.475 * 10^10^	4.637 * 10^10^	3.789 * 10^10^
3 *10^14^	1.31 * 10^11^	7.038 * 10^10^	1.391 * 10^11^	8.704 * 10^10^
5 * 10^14^	2.19 * 10^11^	9.240 * 10^10^	2.318 * 10^11^	1.197 * 10^11^
7.5 * 10^14^	3.28 * 10^11^	1.131 * 10^11^	3.478 * 10^11^	1.492 * 10^11^
10^15^	4.37 * 10^11^	1.275 * 10^11^	4.637 * 10^11^	1.716 * 10^11^
5 * 10^15^	2.19 * 10^12^	2.122 * 10^11^	2.318 * 10^12^	3.100 * 10^11^

*IMBA* Integrated Modules for Bioassay Analysis (based on the ICRP model with first-order kinetics); model of Rump et al. (2019) (modified ICRP model with Michaelis–Menten kinetics for thyroidal uptake of iodide); pregnancy model by Berkovski (2002) at pregnancy week 0, i.e., non-pregnant state (multiple compartment model with first-order kinetics); new pregnancy model at pregnancy week 0 (Berkovski model modified using Michaelis–Menten kinetics for all iodide transport processes involving the NI symporter except iodide intestinal absorption)

For activities exceeding 10^14^ Bq, equivalent thyroid doses continue to increase linearly with the activity in the models based on first-order kinetics (IMBA, original Berkovski model), while the curves flatten out with increasing activity when using the models with integrated NI symporter (model of Rump et al, 2019 and adapted Berkovski model) as an expression of the saturable transport mechanism through the membrane (Fig. 3, Table 5). For an activity of 5*10^15^ Bq, the doses calculated with the models using Michaelis–Menten kinetics are roughly ten times lower than when using first-order kinetics (Table 5). Although in the new pregnancy model, thyroid equivalent doses are numerically higher than in our previously developed model, the values are nevertheless in the same order of magnitude (e.g., for 5*10^15^ Bq: 2.1*10^11^ vs. 3.1*10^11^ mSv) (Table 5).

Iodine-131 activities showing saturation of the uptake mechanism by flattening of the curves (> 10^14^ Bq) are well above the ranges that play a role in nuclear medicine. For radioiodine ablation of post-surgical residues in patients with thyroid cancer, generally an administration of several GBq is used (Hackshaw et al. 2007; Lassmann et al. 2010). During the “Castle Bravo” nuclear weapon test accident on the Marshall Islands (1954), an average thyroid equivalent dose of 7.6 Gy was reconstructed for the victims on the Rongelap Atoll who were most severely affected by early fallout (Simon et al. 2010), corresponding to an incorporated iodine 131 activity of approximately 20 MBq. These activities are clearly in the linear part of all the models, and for practical purposes first-order kinetics can approximately be assumed (Fig. 3). However, for iodine amounts of 100 mg (corresponding to the recommended amount of stable iodine for thyroid blocking), the saturation of the uptake mechanism must be taken into account, as shown by the flattened curves for the models with the integrated NI–symporter (Fig. 3).

**Fig. 4:**
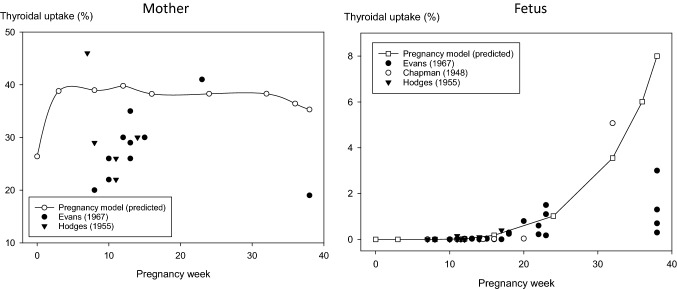
Fractional thyroidal iodide uptake after 24 h (% of an acute iodine intake by the mother) depending on pregnancy/fetal age as predicted by our new model or empirically determined in humans. Source of the data: Chapman et al. 1948; Hodges et al. 1955; Evans et al. 1967

According to our model, the thyroidal iodide uptake in the maternal gland without thyroid blocking increases during the first weeks of pregnancy from roughly 25 to 40% to remain quite stable in the further course (Fig. 4). Empirically measured values of thyroidal iodide uptake in pregnant women show a broad range (20–50%), framing our predictions. Most measured values are below the predictions of our model (Fig. 4). It must be acknowledged that most empirical measurements were done in the first half of pregnancy, whereas the database is very sparse thereafter for understandable ethical reasons.

According to our model, thyroidal iodide uptake by the fetal gland without thyroid blocking disproportionately increases after the 16th week of pregnancy. The empirically measured uptake values correspond quite well to the predictions, except at term (Fig. 4). This latter finding is not really surprising, as only glands from fetuses with non-survivable malformations (anencephalic fetuses) could be examined for ethical reasons at this time point. Similar to the maternal findings, most empirical values originate from the pregnancy period up to the 24th pregnancy week and data are sparse after that. The excellent match of the values predicted by the original model of Berkovski and measured values has already been described (Berkovski 2002). Still, we were able to include an additional set of empirical data from the literature.

#### Maternal and fetal thyroid equivalent doses over pregnancy without thyroid blocking

**Fig. 5:**
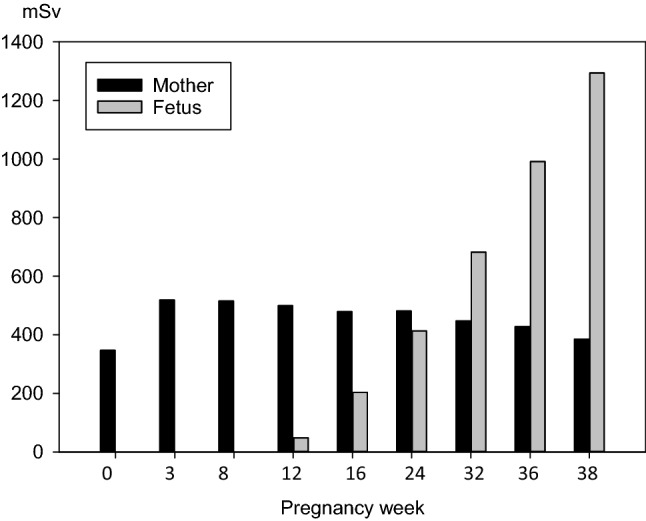
Thyroid equivalent doses in the mother and fetus resulting from an acute iodine 131 intake of 700,000 Bq as predicted by our model at different ages of pregnancy

At pregnancy week 0, the thyroid equivalent dose after the intake of 700,000 Bq iodine 131 in the maternal gland amounts to 347 mSv according to our model and thus is in the range expected from previous calculations for a non-pregnant adult (dose absorbed after 10 days: 336 mSv) (Rump et al., 2019). Within the first weeks of pregnancy, the dose increases to remain in a range between 400 and 520 mSv until term (Fig. 5, Table 6). This is consistent with the enhanced thyroidal uptake fraction for iodide developing during the first pregnancy weeks, as reported in the previous section.

**Table 6:** Protective efficacy of the maternal and fetal thyroid by 100 mg stable iodine against acute radioiodine exposure (700,000 Bq) depending on the week of pregnancy

	Pregnancy week								
	0	3	8	12	16	24	32	36	38
	Mother								
No blocking (mSv)	346.84	518.59	514.97	499.51	478.41	480.94	447.0	427.85	385.55
Blocking without WC (mSv)	190.99	306.76	301.90	292.16	275.97	278.89	268.14	259.86	237.29
Blocking without WC (Eff.)	0.4494	0.4085	0.4178	0.4151	0.4231	0.4201	0.4015	0.3926	0.3845
Blocking with WC (mSv)	3.518	2.900	3.091	3.546	3.095	3.158	2.902	2.884	2.671
Blocking with WC (Eff.)	0.9899	0.9944	0.9940	0.9929	0.9935	0.9930	0.9935	0.9933	0.9931
	Fetus								
No blocking (mSv)	n.a	n.a	n.a	48.06	203.49	412.94	682.59	991.62	1,293.78
Blocking without WC (mSv)	n.a	n.a	n.a	24.42	94.74	194.39	334.73	481.97	623.88
Blocking without WC (Eff.)	n.a	n.a	n.a	0.4919	0.5344	0.5292	0.5096	0.5140	0.5178
Blocking with WC (mSv)	n.a	n.a	n.a	2.850	2.865	2.887	2.899	2.908	2.906
Blocking with WC (Eff.)	n.a	n.a	n.a	0.9407	0.9859	0.9930	0.9958	0.9971	0.9978

Thyroid blockade is initiated at the same time as radioiodine exposure. The efficacy of the iodine blockade is calculated according to the formula Efficacy = 1 – (dose with blockade/dose without blockade). *Eff.* efficacy; *WC* Wolff–Chaikoff effect; *n.a.* not applicable

The fetal thyroid does not accumulate radioiodine before the 10th–12th week of pregnancy (Hodges et al. 1955; Evans et al 1967; Roti et al. 1983; Geoffroy et al. 2000). Iodine uptake remains low up to the 22nd week and rapidly increases thereafter till term (Evans et al. 1967; Geoffroy et al. 2000). From the 12th to the 24th week of pregnancy, the thyroid dose absorbed by the fetal gland is still less than for the maternal gland (12th week: 48 mSv in the fetus, 500 mSv in the mother) (Fig. 5, Table 6). Between the 24th and the 32nd week, the dose absorbed by the fetal thyroid begins to exceed the dose in the maternal gland, and according to our model at term, the equivalent thyroid dose in the fetus amounts to roughly 3 times the value in the mother (Fig. 5, Table 6).

#### Estimation of the protective efficacy of different single doses of stable iodine

**Fig. 6:**
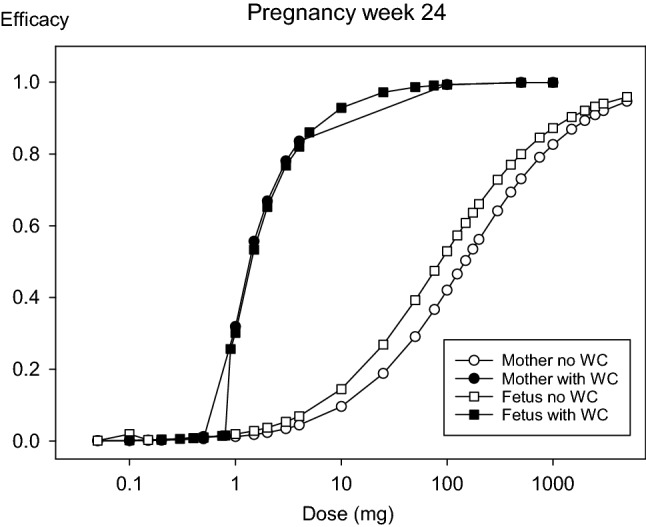
Dose efficacy curves describing thyroidal protection in the mother and fetus in the 24th week of pregnancy for different single doses of stable iodine administered to the mother simultaneously with an acute radioiodine exposure. WC: Wolff–Chaikoff effect

**Fig. 7:**
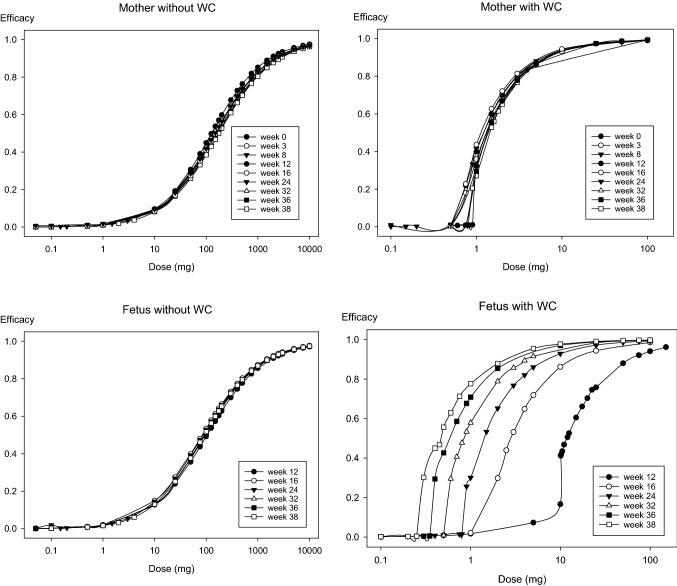
Dose efficacy curves describing thyroidal protection in the mother and fetus at different ages of pregnancy for different single doses of stable iodine administered to the mother simultaneously with an acute radioiodine exposure. WC: Wolff–Chaikoff effect

The calculated thyroidal protection of the maternal and fetal glands increases concomitantly with the amount of single doses of stable iodine administered to the mother simultaneously with acute radioiodine exposure (Figs. 6 and 7). Considering only the competition at the carrier site, the mean effective dose (ED_50_) for protection of the maternal thyroid lies in a range between 125 mg at the beginning of pregnancy and 180 mg at term (Table 7), although in a half-logarithmic plot the dose–effect curves seem to be overlaid (Fig. 7). This is a higher value than previously reported using a simple two-compartment model with integrated NI symporter (ED_50_ = 50.1 mg) (Rump et al. 2019). Considering the Wolff–Chaikoff effect, the mean effective dose is reduced to roughly 1.5 mg all over pregnancy (Table 7). This value is numerically lower, but in the same order of magnitude as previously reported (ED_50_ = 2.70 mg) (Rump et al. 2019). Also, similarly to previous results, the dose–effect curves taking into account the Wolff–Chaikoff effect show a greater steepness, reflected in a higher Hill coefficient compared to the curves describing only the competition at the carrier site (Hill coefficient 2–3 vs. 0.8) (Table 7). As suggested by similar mean effective doses, it seems that the sensitivity of the maternal thyroid toward thyroid blocking remains unchanged over the whole pregnancy if taking into account the Wolff–Chaikoff effect.

**Table 7:** Median effective doses (ED_50_) of the protective efficacy of thyroid blocking by stable iodine and Hill coefficients of the dose–response curves for the mother and fetus at different ages of pregnancy

Pregnancy week	ED_50_ without WC (mg)	Hill coeff	ED_50_ with WC (mg)	Hill coeff	Onset time (min)
Mother					
0	127.6404	0.8599	1.5941	3.1583	15.21
3	154.9070	0.8524	1.2620	2.2510	9.36
8	148.6052	0.8327	1.2847	2.1625	7.85
12	151.0329	0.8351	1.3302	2.1702	7.04
16	145.788	0.8249	1.4168	2.1906	6.44
24	148.6344	0.8231	1.4769	2.0990	5.95
32	162.7179	0.8223	1.5006	2.6205	6.05
36	170.3114	0.8202	1.5479	2.6494	6.22
38	177.4304	0.8222	1.5691	2.7914	6.28
Fetus					
12	104.92	0.7977	12.916	1.9741	126
16	84.7304	0.7934	2.9990	1.9993	42.05
24	87.9998	0.8053	1.6483	2.3657	31.97
32	96.5632	0.8014	0.9320	2.0613	31.82
36	94.8353	0.7174	0.6604	2.7157	30.67
38	93.7058	0.8160	0.4901	2.4510	29.81

Values for the fetus are given from the 12th week on as there is no accumulation of iodine in the fetal thyroid before

*WC* Wolff–Chaikoff effect. The onset times of a total net uptake block of the thyroid caused by the saturation of the gland are given for the usually recommended stable iodine dose of 100 mg

**Fig. 8:**
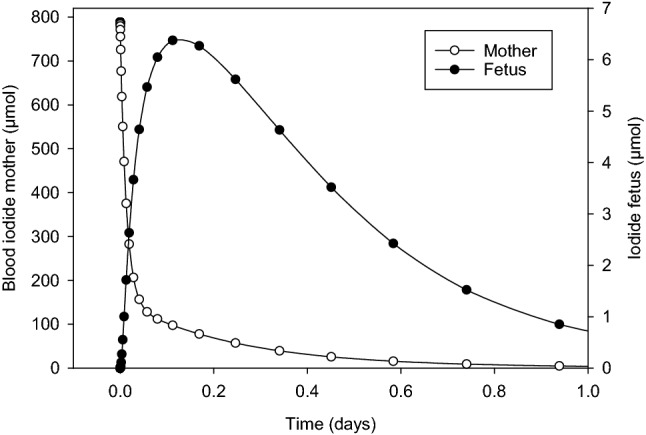
Time course of iodide in the maternal blood compartment and the fetus (represented by a single compartment except for the thyroid) after administration of 100 mg iodide to the mother in the 32nd week of pregnancy

In the case of the fetal thyroid, the mean effective doses for thyroid blocking taking into account only the competition at the carrier site, are numerically lower than in the mother, according to our model (Fig. 6, Table 7). A trend over the different stages of pregnancy is not recognizable (Table 7). The Hill coefficients of the curves are in a similar range as for the mother and quite near unity. Taking into account the Wolff–Chaikoff effect, the mean effective doses show a clear decreasing trend over pregnancy: Starting with a relatively high value of 12.9 mg in the 12th week, the mean effective dose rapidly decreases, reaching 0.5 mg at term (Fig. 7, Table 7). The Hill coefficients are in the same range as for the mother (between 2 and 3). Still, the onset time of the Wolff–Chaikoff effect rapidly and substantially decreases over pregnancy concomitantly with the mean effective doses from 126 min (12th week) to 30 min at term (Table 7). This seems a quite long time compared to the maternal values ranging below 10 min after the 3rd week of pregnancy. Still, it should be considered that in the mother, stable iodide is available in the central blood compartment as soon as medication is administered. In contrast, stable iodide must first reach the fetus and high concentrations be built up, delaying fetal thyroidal iodide uptake (Fig. 8). These findings are quite consistent with the view that the fetal gland is susceptible to thyroid blocking by large doses of stable iodine.

#### Estimation of thyroidal protection by the recommended stable iodine dose

**Fig. 9:**
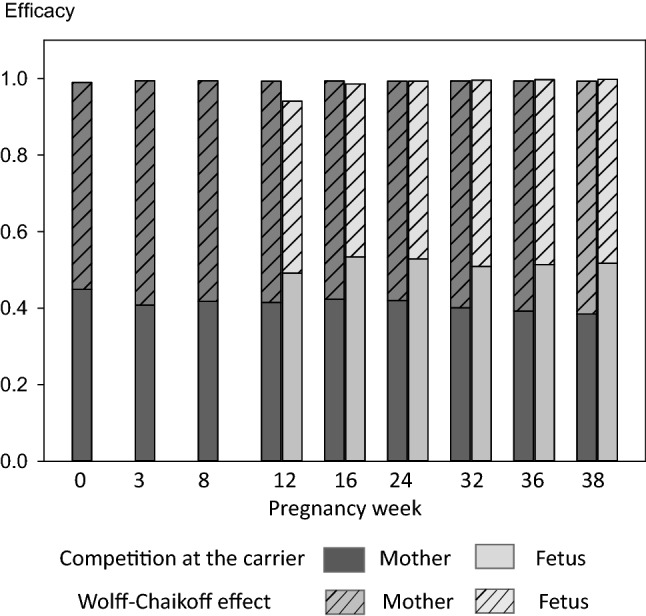
Efficacy of thyroidal protection in the mother and fetus achieved by 100 mg stable iodine administered to the mother simultaneously with an acute radioiodine exposure at different pregnancy ages. Total efficacy results from the combination of competition at the carrier site in the thyrocyte membrane and the Wolff–Chaikoff (WC) effect

The administration of 100 mg stable iodine to the mother at the time of radioiodine exposure will confer very satisfactory protection to the maternal as well as the fetal thyroid in all stages of pregnancy (Fig. 9, Table 6). In the mother, protective efficacy reaches or exceeds 99%. Efficacy of thyroidal protection in the fetus amounts to 94% in the 12th week of pregnancy and increases over time to exceed 99% at term.

Thyroidal protection is due to the combination of the competition of stable iodide and radioiodide at the NI symporter and the Wolff–Chaikoff effect. The contribution of the competition mechanism at the membrane to the total protection is in the range of 40% in the maternal and of 50% for the fetal gland (Fig. 9, Table 6). Thus, at the difference to previous simulations performed with models for non-pregnant adults, the competition mechanism is less prominent in the current pregnancy model (40 vs. 66%) (Rump et al. 2019), although total thyroidal protection is highly satisfactory.

## Discussion

Most organ systems show progressive physiological changes during pregnancy (Pariente et al. 2016). In particular, cardiac output and glomerular filtration rate are enhanced and total body water and the plasma volume are increased. Altered activities of drug-metabolizing enzymes like cytochromes P450 isoenzymes or glucuronosyltransferase(UGT)isoenzymes have also been reported (e.g., CYP1A2 is decreased, CYP2B6 and CYP2D6 are increased) (Pariente et al 2016). Thus, for many pharmacological agents, the apparent volumes of distribution are rather enhanced, and protein binding rates in plasma rather decreased with higher free drug concentrations. The renal clearance is also expected to be enhanced, whereas in the case of mainly metabolized agents, the changes of hepatic clearance depend on the enzymes involved. Physiological and pharmacokinetic changes occur progressively during pregnancy, with peak effects during the third trimester. However, many studies do not report trimester-specific results in the same study group. There are categories of medications that are more commonly investigated in pregnancy, particularly medications used to treat chronic diseases (drugs for HIV, antiepileptics) (Pariente et al. 2016). However, the total number of human studies on pharmacokinetics in pregnancy is limited, as pregnant women are often not included in drug trials for safety and ethical reasons (Rodger et al. 2003; McCullough et al. 2005), so the findings are sometimes conflicting (Pariente et al. 2016). Nevertheless, compartmental physiologically based pharmacokinetic models describing the kinetics of pregnant women have been set up and, in part, validated in comparison to empirically measured drug concentrations (Gaohua et al. 2012). These models are sometimes quite complex, e.g., with 26 maternal and 15 embryo/fetal compartments (Luecke et al. 1994). The latter model is formulated as a set of 41 differential equations leading to computational challenges and the need to select particular iterative optimization algorithms. Other simpler models (e.g., Gaohua et al. 2012) focus on maternal drug exposure and represent the fetoplacental unit only by a single compartment, so that meaningful concentration estimates for the fetus cannot be made. As a departure for our study, we selected a model specifically developed to describe the kinetics of low amounts of iodine and parametrized for different stages of gestation. Although this model is made of a total of 21 compartments (14 maternal and 7 fetoplacental compartments), we had to duplicate it to be able to describe the kinetics of radioiodide and stable iodide separately and to connect the two submodels to simulate the competition at the NI symporter site. For the complex computations, we used the Rosenbrock integration method with simulation results given with variable time intervals, so that we had in part to linearly interpolate the values of interest between two given simulation points with an associated loss of precision.

In the model, we replaced the first-order kinetics by Michaelis–Menten kinetics for transport processes known to be mediated by the NI symporter. As we derived the maximum transport capacity (*T*_max_) from the first-order kinetics rate constants and the volumes of distribution as given by Berkovski (2002), the original model and our adapted model are mathematically equivalent for (radio)iodide concentrations far below the Michaelis–Menten constant (*K*_m_). Compared to simulation results using our previously developed simple two-compartment thyroid blocking model (Rump et al. 2019), the competition between stable iodide and radioiodide at the membrane carrier site of the maternal thyroid seems to be somewhat less effective in the present model (pregnancy week 0, i.e., non-pregnant state without Wolff–Chaikoff effect ED_50_ = 127.6 mg vs. ED_50_ = 50.1 mg in our previous study) (Rump et al. 2019). At first sight, this is not really surprising, as a relatively large distribution volume of 7 l for the central blood compartment was assumed for the whole pregnancy, including week 0. However, taking into account the rate constant (k), the resulting thyroidal iodide clearance is well in the physiological range for a non-pregnant adult (Cl = k * V = 2.3 d^−1^ * 7 l = 11.2 ml min^−1^). Another explanation that should be considered is that due to the numerous additional compartments that are in exchange with the central blood compartment, and the gastrointestinal cycle in the present model, a reduced amount of stable iodide remaining in the central blood compartment may be expected. On the other hand, taking into account the Wolff–Chaikoff effect, in comparison to our previous model (Rump et al. 2019), the potency of stable iodine for the protection of the maternal thyroid is numerically even higher in the present model (ED_50_ = 1.59 mg vs. ED_50_ = 2.70). This is related to the earlier onset of a total net uptake block for iodide by the Wolff–Chaikoff effect (15.2 min vs. 39.5 min in our previous study) (Rump et al. 2019). Despite these numerical deviations, the computed mean effective doses for protecting the maternal thyroid in a non-pregnant woman are in the same order of magnitude. The differences may well be just the consequence of the different structures and parameters of the current and previous models.

The changes of the rate constant for the transfer of iodide from the blood into the organic iodine compartment of the thyroid over the course of pregnancy as given in the original model of Berkovski (e.g., 2.3 d^−1^ at week 0, 9.3 d^−1^ at week 24), corresponding to a well-known increase of thyroidal iodide clearance, are reflected in our model by parallel deviations of the maximum transport capacity. According to our model, considering only the competition at the carrier site, the protective potency of stable iodine is less at term compared to earlier stages of pregnancy. On the other hand, taking into account the Wolff–Chaikoff effect, the sensitivity of the maternal thyroid seems to remain stable over pregnancy, as reflected by the comparable mean effective doses of stable iodine.

The predicted maternal and fetal thyroidal uptake fractions for iodide estimated with our adapted pregnancy model are also in the same order of magnitude as the values empirically measured at different pregnancy ages. This has already been shown for the original Berkovski model for the fetal thyroid. Still, we were able to add another empirical data set (Chapman et al. 1948) to confirm this statement further. As our objective was to study not just radioiodine kinetics but thyroidal blocking, we had to consider the contribution of the Wolff–Chaikoff effect. In analogy to a previous model consistent with empirical efficacy data, we modeled this effect by a transient 36 h total net uptake block for iodide into the thyroid when the gland is saturated (“switch on, switch off” model). Saturation amounts have been reported for adults, and for the maternal thyroid, we used the value given by Ramsden et al. (1967) (+ 350 µg above the basic content of 8000 µg). As we are not aware of published saturation amounts for the fetal thyroid, we assumed that saturation is reached when an amount corresponding to the same fraction of the iodine content as in the adult (+ 4.375%) has been taken up.

The validity of this assumption is closely related to the question of the sensitivity of the fetal thyroid gland to unphysiologically high iodine intake, e.g., through high intake with food or through the consumption of dietary supplements by the mother. The bulk of the literature is focused on the impossibility of the fetal thyroid to escape the Wolff–Chaikoff effect and thus the prolonged thyroid blocking. Animal experiment results are contradictory, even in the same species, e.g., rats (Lebsir et al. 2019; Gaouaoui-Azouaou et al. 2022), and clinical experience does not allow to solve this issue (Markou et al. 2001). Although the duration of thyroid blocking in the fetus is of great importance because of its possible negative impact on fetal development, the extended time line is not a major issue for the results of our simulations, as we consider only acute radioiodine exposure with a simultaneous single administration of stable iodine. Much more important for our results is the saturation amount in the fetal thyroid determining the “switch on” time of the Wolff–Chaikoff effect. There are even less information on this value than on the extended duration of the Wolff–Chaikoff effect, so that we had to rely on the (fraction) value given for the mature adult gland as the best available estimate.

We are fully aware that this assumption may be challenged easily: the turnover of iodine in the fetal thyroid is much more rapid than in an adult gland as also reflected in the original model of Berkovski with organic iodine being secreted from the fetal thyroid with a half-life of 19.8 d (= ln 2 /0.035 d^−1^) compared with 110 d (= ln 2/0.0063 d^−1^) in the mother at week 0 of pregnancy. Even shorter biological half-lives for iodine of only a few days have been given for the fetal thyroid (National Cancer Institute 2015). Moreover, there are strong indications that the total iodine amount in the gland is not the cybernetic variable actually governing thyroidal iodide uptake, so even the term “saturation” is probably erroneous. It seems that there is an inverse relation between the transport capacity through the basolateral membrane and the “saturation” amount, as shown when comparing iodide pharmacokinetics in Caucasians and Japanese with a high nutritional daily iodine intake (Rump et al. 2021b). However, as describing thyroidal protection is not meaningful if disregarding the Wolff–Chaikoff effect and, given the lack of information and data on the fetal thyroid, we did not see an alternative to model this effect using relative saturation values analogous to the adult gland.

Compared to the maternal gland, the situation for the fetal thyroid is much more complex. Radiation exposure through the accumulation of radioiodine by the fetal thyroid can only be assumed from about the 12th pregnancy week with initially a relatively low equivalent dose absorbed. The initially low radioiodine accumulation then increases continuously over pregnancy. Whereas the equivalent dose in the 12th week is still under the load on the maternal gland without thyroid blocking measures, the dose absorbed by the fetal gland at term is about 3 times that of the maternal gland. This result of our simulations is in good agreement with the values described in the literature. Whereas the relative iodine 131 concentration ratio of the fetal relative to the maternal gland is very low in the first trimester, it exceeds unity in late gestation with a midrange of about 5 (National Cancer Institute 2015).

In the case of thyroid blocking, the mean effective doses for the competition at the carrier site alone in the fetal thyroid are much lower than for the maternal gland. Still, they remain in the same order of magnitude from the 16th week of pregnancy to term. The situation is very different when taking into account the Wolff–Chaikoff effect. The protective potency for the fetal thyroid is substantially less in the 12th pregnancy week than for the maternal gland (ED_50_ = 12 mg vs. 1.5 mg). In the further course, there is a continuing shift of the dose–effect curves to the left with the highest sensitivity of the fetal thyroid at term (ED_50_ = 500 µg). Due to the increasing maximum transport capacity through the thyrocyte membrane, it is understandable that the saturation threshold is reached faster and faster during pregnancy. So the total net uptake stop for radioiodide becomes effective more rapidly. This corresponds well to the view that the fetal thyroid is particularly sensitive to large iodine amounts. The dosages necessary to block the fetal gland are still several times above the recommended (200 µg) and usual daily nutritional iodine intakes. Still, our results seem well in line with recommendations in the literature to avoid excess iodine intake by the mother, as it could have a negative effect on the development of the fetus by inhibiting thyroid function. However, it was shown that in populations with a very rich iodine diet, e.g., in Japan, thyroidal iodide uptake is down-regulated (Matsunaga et al. 2001; Rump et al. 2021b). This might possibly be also the case for the fetal thyroid in the case of a high daily nutritional iodine intake by the mother. But, as far as we know, there are no kinetic data available on this point.

An important issue is to know whether the dosages of stable iodine for thyroid blocking should be adapted during pregnancy. Official guidelines recommend an unchanged dosage of 100 mg of stable iodine for pregnant women as for other adults. Our simulation results clearly confirm that this dosage is sufficient for good protection of the maternal and fetal thyroid in all stages of pregnancy in the case of acute radioiodine exposure. These results are in line with experimental findings on chimpanzees showing that the 24 h uptake of iodine-123 in the fetal thyroid was satisfactorily reduced by iodine dosages similar to those recommended in humans (Noteboom et al. 1997). Despite differences in thyroid hormone metabolism between humans and apes that may even contribute to the different trajectories of brain development in the two species (Gagneux et al. 2001; Varki et al. 2005), monkeys are considered to be the best available model for human thyroid function assessment (Pickering 1964; Ozpinar et al. 2011).

It was previously shown that a single dose of stable iodine in adults does not confer satisfactory thyroidal protection in the case of prolonged radioiodine exposure as must be expected in power plant accidents (Imanaka et al. 2015; Rump et al. 2019; Eder et al. 2020). To achieve a satisfactory thyroidal protection, repetitive stable iodine administrations are required (Rump et al. 2019; Eder et al. 2020). The WHO does not give clear guidelines on this issue, although it is acknowledged that repetitive thyroid blocking may be necessary (WHO 2017). Preclinical data indicate that repetitive daily dosages of 1 mg/kg for 8 days do not induce adverse effects in adult rats (Lebsir et al. 2018a, b) and this is consistent with clinical experience: Lugol’s solution has been used for a long time in patients planned for thyroidectomy and historically Plummer used 80–320 mg daily for 10 days as a well-established treatment (Plummer 1924). Even higher daily dosages up to 800 mg daily have been reported in the literature (Okamura et al. 2014; Calissendorf et al. 2017).

Although the issue of prolonged radioiodine exposure and repetitive thyroid blocking were not examined in the present analysis, it is probable that a single dose of stable iodine will not confer satisfactory protection to the maternal or fetal thyroid. However, the situation in pregnant women is highly complex as thyroid blocking in the unborn child may have negative impacts like the induction of hypothyroidism and neurocognitive development impairment (Markou et al. 2001). In a pregnant rat model, it was shown that repetitive stable iodine administrations are associated with an impairment of neurocognitive development (Lebsir et al. 2019) and with modified gene expression profiles in the thyroid and brain cortex of the progeny after birth (Cohen et al. 2020). Other experimental studies do not confirm thyroidal toxicity precluding repetitive stable iodine administrations in pregnancy (Noteboom et al. 1997; Gaouaoui-Azouaou et al. 2022). It was stated that the ability to fully escape from the acute Wolff–Chaikoff effect in man does not mature until the 36th week of pregnancy (Pearce et al. 2016). On the other hand, experiences following the Chernobyl accident 1986 showed that only 0.37% among neonates of mothers having got stable iodine for thyroid blocking (12 from a sample of 3214 newborns) had signs of hypothyroidism at birth and the anomalies have been reported to have disappeared 16–20 days after birth (Naumann et al. 1993; Verger et al. 2001). In a clinical case of fetal hypothyroidism goiter diagnosed in the 21st gestation week due to a continuous excess consumption of supplements containing iodine, the mother was given 100 µg of levothyroxine daily, and in addition three intra-amniotic levothyroxine injections were performed till the 26th week. Following the last levothyroxine injection, several fetal blood samples were analyzed up to the 30th week to ascertain normal fetal thyroid status. Results suggested that after stopping the excess iodine supplement by the mother, the resumption of fetal thyroid hormone synthesis occurred between the 21st and the 30th week of gestation (Hardley et al., 2018). These findings suggest that the fetal thyroid is able to escape the Wolff–Chaikoff effect (in the reported case in the mid third trimester), even if the timelines might possibly be different from a mature adult gland. However, even mild iodine overloads have been associated with fetal/neonatal hypothyroidism (Sun et al. 2009; Jourdain et al. 2010; Connelly et al., 2012) and even if only transient, this can impair the cognitive development of unborn children (Derksen-Lubsen et al. 1996; Kempers et al. 2006; Jourdain et al. 2010; Overcash et al. 2016). The transfer of thyroxine through the placenta from the mother to the fetus decreases over pregnancy as placental deiodinase activity increases (Girling 2003). Screening at birth with postnatal treatment may not be sufficient to ascertain unimpaired neurodevelopment (Hardley et al. 2018). The WHO (2017) recommends that pregnant women should not receive repeated stable iodine doses due to the risk of adverse effects. The issue of repetitive stable iodine administrations in the case of prolonged radioiodine exposure in pregnant women remains thus unresolved and needs research to develop an adapted iodine blockade strategy. Although considered an agent of second choice in official recommendations, the use of perchlorate for thyroid blocking should be revisited. In pregnancy, it could be advantageous because of its simpler pharmacological mechanism without Wolff–Chaikoff effect.

## Conclusion

The possibilities of experimental studies on thyroid blocking in pregnant women are extremely limited for ethical reasons. So, results from animal studies with all the uncertainties associated with the translation of data to humans or indirect clinical findings must be used to improve our knowledge. Simulations based on biokinetic and dosimetric models may be an additional tool to draw conclusions about health hazards in uncommon emergencies like nuclear or radiological incidents and to develop therapeutic strategies. The real challenge is to create models with a valid structure and in particular valid parameters. We departed from a pregnancy model for radioiodine that is well compatible with empirical findings and adapted it to be able to simulate thyroid blocking. It seems that this model could be valuable to give at least estimates of health hazards in case of radioiodine exposure and the efficacy of large doses of stable iodine in the mother and fetus. Our simulations in this study were limited to acute radioiodine exposure and a single dose of stable iodine. As in the case of power plant accidents, prolonged radioiodine exposures must be expected, more complex scenarios with different repetitive stable iodine dosage schemes and the study of other protective agents, like, e.g., perchlorate, need further assessment.

## References

[CR1] Aboul-Khair SA, Crooks J, Turnbull AC, Hytten FE (1964). The physiological changes in thyroid function during pregnancy. Clin Sci.

[CR2] Aboul-Khair SA, Buchanan TJ, Crooks J, Turnbull AC (1966). Structural and functional development of the human foetal thyroid. Clin Sci.

[CR3] Adams CA, Bonnell JA (1962). Administration of stable odide as a means of reducing thyroid irradiation resulting from inhalation of radioactive iodine. Health Phys.

[CR4] Agency for Toxic Substances and Disease Registry (ATDSR)(2004) Toxicological profile for iodine. US Department of Health and Human Services, Public Health Service, Atlanta 38091462

[CR5] Autorité de Sureté Nucléaire (ASN)(2008) Guide national. Intervention médicale en cas d´évènement nucléaire ou radiologique. Version V 3.6.

[CR6] Barbosa RM, Andrade KC, Silveira C, Almeida CM, Souza RT, Oliveira PF, Cecatti JG (2019). Ultrasound measurements of fetal thyroid: Reference ranges from a cohort of low-risk pregnant women. BioMed Res Int.

[CR7] Berkovski V (1999a) Radioiodine biokinetics in the mother and fetus. Part 1. Pregnant woman. In: Radiation and Thyroid Cancer. Publication No. EUR 18552 EN. World Scientific Publishing, London, pp 319–325

[CR8] Berkovski V (1999b) Radioiodine biokinetics in the mother and fetus. Part 2. Fetus. In: Radiation and Thyroid Cancer. Publication No. EUR 18552 EN. World Scientific Publishing, London, pp 327–332

[CR9] Berkovski V (2002). New iodine models family for simulation of short-term biokinetic processes, pregnancy and lactation. Food Nutr Bull.

[CR10] Birchall A, Puncher M, Marsh JW, Davis K, Bailey MR, Jarvis NS, Peach AD, Dorrian MD, James AC (2007). IMBA Professional Plus: a flexible approach to internal dosimetry. Radiat Prot Dosimetry.

[CR11] Blum M, Eisenbud M (1967). Reduction of thyroid irradiation from 131 I by potassium iodide. JAMA.

[CR12] Bürgi H (2010). Iodine excess. Best Pract Res Clin Endocrinol Metab.

[CR13] Calissendorff J, Falhammar H (2017). Lugol's solution and other iodide preparations: perspectives and research directions in Graves disease. Endocrine.

[CR14] Chabot G (2016) Radiation basics. Fission, Fusion. Answer to question Q10097 - Which fission products are present in a nuclear reactor? Various sources say “many” or hundreds and then refer to 131 I, 137 Cs, and 90Sr as important products in the case of exposure. Health Physics Society. https://hps.org/publicinformation/ate/q10097.html. Accessed 09 Aug 2021

[CR15] Chapman EM, Corner GW, Robinson D, Evans RD (1948). The collection of radioactive iodine by the human fetal thyroid. J Clin Endocrinol Metab.

[CR16] Chou TC, Talaly P (1977). A simple generalized equation for the analysis of multiple inhibitions of Michaelis-Menten kinetic systems. J Biol Chem.

[CR17] Cohen DPA, Benadjaoud MA, Lestaevel P, Lebsir D, Benderitter M, Souidi M (2020) Effects of repetitive Iodine thyroid blocking on the foetal brain and thyroid in rats: a systems biology approach. Sci Rep 10:10839. https://www.nature.com/articles/s41598-020-67564-8.pdf. Accessed 09 Aug 2021 10.1038/s41598-020-67564-8 PMC7331645 32616734

[CR18] Connelly KJ, Boston BA, Pearce EN, Sesser D, Snyder D, Braverman LE, Pino S, LaFranchi SH (2012). Congenital hypothyroidism caused by excess prenatal maternal iodine ingestion. J Pediatr.

[CR19] Darrouzet E, Lindenthal S, Marcellin D, Pellequer JL, Pourcher T (2014). The sodium/iodide symporter: State of the art of its molecular characterization. Biochim Biophys Acta.

[CR20] Derksen-Lubsen G, Verkerk PH (1996). Neuropsychologic development in early treated congenital hypothyroidism: analysis of literature data. Pediatric Res.

[CR21] Eder S, Hermann C, Lamkowski A, Kinoshita M, Yamamoto T, Abend M, Shinomiya N, Port M, Rump A (2020). A comparison of thyroidal protection by stable iodine or perchlorate in the case of acute or prolonged radioiodine exposure. Arch Toxicol.

[CR22] Evans TC, Kretzschmar RM, Hodges RE, Song CW (1967). Radioiodine uptake studies of the human fetal thyroid. J Nucl Med.

[CR23] Feely J (1979). The physiology of thyroid function in pregnancy. Postgrad Med J.

[CR24] Gagneux P, Varki A (2001). Genetic differences between humans and great apes. Mol Phylogenet Evol.

[CR25] Gaohua L, Abduljalil K, Jamei M, Johnson TN, Rostami-Hodjegan A (2012). A pregnancy physiologically based pharmacokinetic (p-PBPK) model for disposition of drugs metabolized by CYP1A2, CYP2D6 and CYP3A4. Br J Clin Pharmacol.

[CR26] Gaouaoui-Azouaou H, L’Homme B, Benadjaoud MA, Sache-Aloui A, Granger R, Voyer F, Lestaevel P, Gruel G, Caire-Maurisier F, Crambes C, Dare-Doyen S, Benderitter M, Souidi M (2022) Protection and safety of a repeated dosage of KI for iodine thyroid blocking during pregnancy. J Radiol Prot 42(1). doi: 10.1088/1361-6498/ac336e. https://iopscience.iop.org/article/10.1088/1361-6498/ac336e. Accessed 01 June 2022 10.1088/1361-6498/ac336e 34700314

[CR27] Geoffroy B, Verger P, Le Guen B (2000). Pharmacocinétique de l’iode: revue des connaissances utiles en radioprotection accidentelle. Radioprotect.

[CR28] Girling JC (2003). Thyroid disorders in pregnancy. Cur Obstet Gynaecol.

[CR29] Glinoer D, De Nayer P, Bourdoux P, Lemone M, Robyn C, Van Steirteghem A, Kinthaert J, Lejeune B (1990). Regulation of maternal thyroid during pregnancy. J Clin Endocrinol Metab.

[CR30] Hackshaw A, Harmer C, Mallick U, Haq M, Franklyn JA (2007). 131I activity for remnant ablation in patients with differentiated thyroid cancer: a systematic review. Rev J Clin Endocrinol Metab.

[CR31] Hall SC, Suresh S (2016) Neonatal anesthesia. In: anesthesia key. Fastest Anesthesia & Intensive Care & Emergency Medicine Insight Engine. https://aneskey.com/neonatal-anesthesia/. Accessed 09 Aug 2021

[CR32] Hardley MT, Chon AH, Mestman J, Nguyen CT, Geffner ME, Chmait RH (2018). Iodine-induced fetal hypothyroidism: diagnosis and treatment with intra-amniotic levothyroxine. Horm Res Paediatr.

[CR33] Harvey RP, Hamby DM, Palmer TS (2004). A modified ICRP 66 iodine gas uptake model and its parametric uncertainty. Health Phys.

[CR34] Hine G, Brownell G (1956). Radiation dosimetry.

[CR35] Hodges RE, Evans TC, Bradbury JT, Keettel WC (1955). The accumulation of radioactive iodine by human fetal thyroids. J Clin Endocrinol Metab.

[CR36] Horn-Lodewyk J (2019). Correlation of radioiodine doses for 6-h and 24-hour iodine-131 thyroid uptake values for Graves’ hyperthyroidism. Endocrine J.

[CR37] Iglesias ML, Schmidt A, Al Ghuzlan A, Lacroix L, de Vathaire F, Chevillard S, Schlumberger M (2017). Radiation exposure and thyroid cancer: a review. Arch Endocrinol Metab.

[CR38] Imanaka T, Hayashi G, Endo S (2015). Comparison of the accident process, radioactivity release and ground contamination between Chernobyl and Fukushima-1. J Radiat Res.

[CR39] International Commission on Radiological Protection (ICRP) (2001) Doses to the embryo and fetus from intakes of radionuclides by the mother. ICRP publication 88. Ann ICRP 31(1–3), corrected version, May 2002. https://www.icrp.org/publication.asp?id=ICRP%20Publication%2088. Accessed 09 Aug 2021 10.1016/S0146-6453(01)00022-7 11730884

[CR40] Johansson L, Leide-Svegborn S, Mattsson S, Nosslin B (2003). Biokinetics of iodide in man: refinement of current ICRP dosimetry models. Cancer Biother Radiopharm.

[CR41] Jourdain JR, Herviou K (2010) Medical effectiveness of iodine prophylaxis in a nuclear reactor emergency situation and overview of European practices. Radiation Protection No 165. Final report of contract TREN/08/NUCL/SI2.520028. Publications Office of the European Union, Luxemburg

[CR42] Kempers MJ, van der Sluijs VL, Nijhuis-van der Sanden MW, Kooistra L, Wiedijk BM, Faber I (2006). Intellectual and motor development of young adults with congenital hypothyroidism diagnosed by neonatal screening. J Clin Endocrinol Metab.

[CR43] Kiserud T, Piaggio G, Carroli G, Widmer M, Carvalho J, Neerup Jensen L, Giordano D, Cecatti JG, Aleem HA, Talegawkar SA, Benachi A, Diemert A, Tshefu Kitoto A, Thinkhamrop J, Lumbiganon P, Tabor A, Kriplani A, Perez RG, Hecher K, Hanson MA, Gülmezoglu AM, Lawrence D. Platt LD (2017). The World Health Organization fetal growth charts: a multinational longitudinal study of ultrasound biometric measurements and estimated fetal weight. PLOS Med 14(1): e1002220. https://journals.plos.org/plosmedicine/article?id=10.1371/journal.pmed.1002220. Accessed 09 Aug 2021 10.1371/journal.pmed.1002220 PMC5261648 28118360

[CR44] Kovari M (1994). Effect of delay time on effectiveness of stable iodine prophylaxis after intake of radioiodine. J Radiol Prot.

[CR45] Lassmann M, Reiners C, Luster M (2010). Dosimetry and thyroid cancer: the individual dosage of radioiodine. Endocr Relat Cancer.

[CR46] Lebsir D, Manens L, Grison S, Lestaevel P, Ebrahimian T, Suhard D, Phan G, Dublineau I, Tack K, Benderitter M, Pech A, Jourdain JR, Souidi M (2018). Effects of repeated potassium iodide administration on genes involved in synthesis and secretion of thyroid hormone in adult male rat. Mol Cell Endocrinol.

[CR47] Lebsir D, Cohen David, Manens L, Grison S, Tack K, Benderitter M, Pech, Lestaevel P, Soouidi M (2018b) Toxicology of repeated iodine thyroid blocking in adult rat. J Pharm Res 3(1):1–8. https://www.researchgate.net/publication/325169797_Toxicology_of_Repeated_Iodine_Thyroid_Blocking_in_Adult_Rat/link/5b4337f70f7e9bb59b18158b/download. Accessed 09 Aug 2021

[CR48] Lebsir D, Guemri J, Kereselidze D, Grison S, Benderitter M, Pech A, Cohen D, Benadjaoud MA, Lestaevel P, Souidi M (2019). Repeated potassium iodide exposure during pregnancy impairs progeny’s brain development. Neuroscience.

[CR49] Leung AM, Braverman LE (2014). Consequences of excess iodine. Nat Rev Endocrinol.

[CR50] Luecke RH, Wosilait WD, Pearce BA, Young JF (1994). A physiologically based pharmacokinetic computer model for human pregnancy. Teratol.

[CR51] Macey R, Oster G, Zahnley T (2009) Berkeley Madonna user's guide. Version 8.02. University of California, Department of Molecular and Cellular Biology, Berkeley CA

[CR52] Marinelli L, Quimby E, Hine G (1948). Dosage determination with radioactive isotopes: II. Practical considerations in therapy and protection. Am J Roentgenol Radium Ther Nucl Med.

[CR53] Markou K, Georgopoulos N, Kyriazopoulou V, Vagenakis AG (2001). Iodine-induced hypothyroidism. Thyroid.

[CR54] Matsunaga T, Kobayashi K (2001). Sensitivity analysis on the deposition of inhaled radioactive iodine and the effectiveness of iodine prophylaxis. Jpn J Health Phys.

[CR55] McCullough LB, Coverdale JH, Chervenak FA (2005). A comprehensive ethical framework for responsibly designing and conducting pharmacologic research that involves pregnant women. Am J Obstet Gynecol.

[CR56] National Cancer Institute (2015) Estimated exposure and thyroid doses report. https://www.cancer.gov/about-cancer/causes-prevention/risk/radiation/i131-report-and-appendix. Accessed 09 Aug 2021

[CR57] Nauman J, Wolff J (1993). Iodide prophylaxis in Poland after the Chernobyl reactor accident: benefits and risks. Am J Med.

[CR58] Nicola JP, Basquin C, Portulano C, Reyna-Neyra A, Paroder M, Carrasco N (2009). The Na+/I- symporter mediates active iodide uptake in the intestine. Am J Physiol Cell Physiol.

[CR59] Noteboom JL, Hummel WA, Broerse JJ, de Vijlder JJM, Vulsma Th, Jansen JThM, van Bekkum DW (1997). Protection of the maternal and fetal thyroid from radioactive contamination by the administration of stable iodide during pregnancy. An experimental evaluation in chimpanzees. Radiat Res.

[CR60] Okamura K, Sato K, Fujikawa M, Bandai S, Ikenoue H, Kitazono T (2014). Remission after potassium iodide therapy in patients with Graves hyperthyroidism exhibiting thionamide-associated side effects. J Clin Endocrinol Metab.

[CR61] Overcash RT, Krishelle LMA, Hull AD, Ramos GA (2016). Maternal iodine exposure: A case of fetal goiter and neonatal hearing loss. Pediatrics.

[CR62] Ozpinar A, Golub MS, Poppenga RH, Blount BC, Gillespie JR (2011). Thyroid status of female rhesus monkeys and preliminaryinformation on impact of perchlorate administration. Lab Anim.

[CR63] Pariente G, Leibson T, Carls A, Adams-Webber T, Ito S, Koren G (2016) Pregnancy-associated changes in pharmacokinetics: A systematic review. PLoSMed 13(11):e1002160. https://journals.plos.org/plosmedicine/article?id=10.1371/journal.pmed.1002160. Accessed 09 Aug 2021 10.1371/journal.pmed.1002160 PMC5089741 27802281

[CR64] Pearce EN, Lazarus JH, Moreno-Reyes R, Zimmermann MB (2016). Consequences of iodine deficiency and excess in pregnant women: an overview of current knowns and unknowns. Am J Clin Nutr.

[CR65] Pickering DE (1964). Maternal thyroid hormone in the developing fetus. Observations on monkeys (*Macaca mulatta*). Am J Dis Child.

[CR66] Plummer HS (1924). The value of iodine in exopthalmic goiter. J Iowa Med Soc.

[CR67] Ramsden D, Passant FH, Peabody CO, Speight RG (1967). Radioiodine uptakes in the thyroid studies of the blocking and subsequent recovery of the gland following the administration of stable iodine. Health Phys.

[CR68] Ratnakar Rao N, Shetty Patil B (2015). Histological study of thyroid gland among fetus in different age groups. Int J Biol Med Res.

[CR69] Reiners C, Schneider R (2013). Potassium iodide (KI) to block the thyroid from exposure to I-131: current questions and answers to be discussed. Radiat Environm Biophys.

[CR70] Reiners C, Drozd V, Yamashita S (2020). Hypothyroidism after radiation exposure: brief narrative review. J Neural Transmission.

[CR71] Richard K, Li H, Landers KA, Patel J, Mortimer RH (2012) Placental transport of thyroid hormone and iodide. In: Zheng J: Recent advances in research on the human placenta. IntechOpen Ltd, London. https://www.intechopen.com/books/702. Accessed 12 Aug 2021

[CR72] Riggs DS (1952). Quantitative aspects of iodine metabolism. Pharmacol Rev.

[CR73] Rodger MA, Makropoulos D, Walker M, Keely E, Karovitch A, Wells PS (2003). Participation of pregnant women in clinical trials: will they participate and why?. Am J Perinatol.

[CR74] Roti E, Gnudi A, Braverman LE (1983). The placental transport, synthesis and metabolism of hormones and drugs which affect thyroid function. Endocr Rev.

[CR75] Rump A, Stricklin D, Lamkowski A, Eder S, Abend M, Port M (2016). Reconsidering current decorporation strategies after incorporation of radionuclides. Health Phys.

[CR76] Rump A, Eder S, Lamkowski A, Kinoshita M, Yamamoto T, Abend M, Shinomiya N, Port M (2019) Development of new biokinetic-dosimetric models for the simulation of iodine blockade in the case of radioiodine exposure in man. Drug Res 69:583–597. https://www.thieme-connect.com/products/ejournals/abstract/10.1055/a-0960-5590. Accessed 09 Aug 2021 10.1055/a-0960-5590 31390663

[CR77] Rump A, Eder S, Hermann C, Lamkowski A, Kinoshita M, Yamamoto T, Take J, Abend M, Shinomiya N, Port M (2021a) Modeling principles of protective thyroid blocking. Int J Radiat Biol 11:1–12 (online ahead of print). https://www.tandfonline.com/doi/full/. Accessed 07 Mar 2022 10.1080/09553002.2021.1987570 34762000

[CR78] Rump A, Eder S, Hermann C, Lamkowski A, Kinoshita M, Yamamoto T, Abend M, Shinomiya N, Port M (2021b) A comparison of thyroidal protection by iodine and perchlorate against radioiodine exposure in Caucasians and Japanese. Arch Toxicol 95(7):2335–2350. https://www.ncbi.nlm.nih.gov/pmc/articles/PMC8241675/. Accessed 09 Aug 2021b 10.1007/s00204-021-03065-5 PMC8241675 34003340

[CR79] Schäuble S, Stavrum AK, Puntervoll P, Schuster S, Heiland I (2013). Effect of substrate competition in kinetic models of metabolic networks. FEBS Lett.

[CR80] Simon SL, Bouville A, Land CE, Beck HL (2010). Radiation doses and cancer risks in the Marshall Islands associated with exposure to radioactive fallout from Bikini and Enewetak nuclear weapons tests: Summary. Health Phys.

[CR81] Stieve FE, Zemlin G, Griessl I (1985) Placental transfer of iodine and iodine compounds. Contract FE 77552. Gesellschaft für Strahlen- und Umweltforschung GmbH, München Neuherberg

[CR82] Strahlenschutzkommission (SSK) (2018) Verwendung von Jodtabletten zur Jodblockade der Schilddrüse bei einem Notfall mit Freisetzung von radioaktivem Jod. Empfehlung der Strahlenschutzkommission. Verabschiedet in der 294. Sitzung der Strahlenschutzkommission am 26. April 2018. https://www.ssk.de/SharedDocs/Beratungsergebnisse/2018/2018-04-26Jodmerk.html. Accessed 09 Aug 2021

[CR83] Sun XF, Yang XF, Preedy VR, Burrow GN, Watson R (2009). Developmental effects of toxic doses of iodine. Comprehensive handbook of iodine: Nutritional, biochemical, pathological and therapeutic aspects.

[CR84] Takamura N, Nakamura Y, Ishigaki K, Ishigaki J, Mine M, Aoyagi K, Yamashita S (2004). Thyroid blockade during a radiation emergency in iodine-rich areas: effect of a stable-iodine dosage. J Radiat Res (tokyo).

[CR85] Varki A, Altheide TK (2005) Comparing the human and chimpanzee genomes: Searching for needles in a haystack. Genome Res 15: 1746–1758. https://genome.cshlp.org/content/15/12/1746.full.pdf+html. Accessed 09 August 2021 10.1101/gr.3737405 16339373

[CR86] Verger P, Aurengo A, Geoffroy B, Le Guen B (2001). Iodine kinetics and effectiveness of stable iodine prophylaxis after intake of radioactive iodine: a review. Thyroid.

[CR87] World Health Organization (WHO) (2017). Iodine thyroid blocking. Guidelines for use in planning for and responding to radiological and nuclear emergencies. World Health Organization, Geneva 29630192

[CR88] Wolff J, Chaikoff IL (1948). Plasma inorganic iodide as a homeostatic regulator of thyroid function. J Biol Chem.

[CR89] Yoshida S, Ojino M, Ozaki T, Hatanaka T, Nomura K, Ishii M, Koriyama K, Akashi M (2014). Guidelines for iodine prophylaxis as a protective measure: information for physicians. JMAJ.

